# Policy requirements and coach mental health training: a mixed methods analysis of online versus in-person delivery modalities

**DOI:** 10.3389/fspor.2025.1701426

**Published:** 2026-01-09

**Authors:** Samantha Bates, Dawn Anderson-Butcher, Kylee Ault-Baker, Emily Nothnagle

**Affiliations:** College of Social Work, The Ohio State University, Columbus, OH, United States

**Keywords:** mental health, online training, sport coach, sport policy, technology

## Abstract

**Introduction:**

Amid growing concerns about youth mental health, several states in the U.S. have enacted policies requiring mental health training for school-based sport coaches.

**Methods:**

This mixed methods study explored the delivery of mental health training for coaches in Ohio in response to new policy mandates, comparing the effectiveness of in-person and asynchronous online formats. A total of 1,690 coaches completed evaluations after participating in an online (*n* = 978) or in-person (*n* = 712) state-approved mental health training.

**Results:**

Repeated measures ANOVA analyses revealed statistically significant increases in coaches’ confidence in supporting student-athletes with mental health concerns and in linking them to appropriate supports, regardless of the delivery modality (*p* < .001). Small but significant interaction effects indicated slightly greater confidence gains among online participants (*η*^2^ = .011 for supporting concerns; *η*^2^ = .005 for linking to supports). Qualitative analysis of open-ended responses about perceived learning from online participants identified five major themes: (1) Approaching struggling student-athletes, where coaches highlighted new skills in de-escalation, emotional regulation, and engaging in difficult conversations; (2) Wellness check-ins, where coaches learned relational strategies to monitor well-being; (3) Q.P.P.R. (Question, Pause/Persuade, Refer), which improved coaches’ recognition of warning signs and confidence in crisis conversations; (4) Creating dialogue with open-ended questions, which provided sentence starters to elicit meaningful conversations; and (5) Referring student-athletes to resources, which underscored the importance of knowing referral pathways.

**Discussion:**

Findings suggest coach educators can leverage technology to design interactive online coach training sessions that yield comparable learning outcomes to those of in-person training. Our results inform scalable, policy-aligned solutions that can enhance coach preparedness by leveraging technology to equip coaches with best practices in supporting student-athlete mental health.

## Introduction

Rising mental health concerns among young people in the United States (U.S.) have led to significant changes in policy, funding, and resources aimed at better supporting youth in schools and communities. School-based sport, defined as organized education-based athletic programs implemented through schools in the U.S., represents a key setting that has responded to rising mental health concerns among young people by implementing new policies to prepare coaches as allies in mental health promotion and intervention. Increasingly, state agencies overseeing school-based sport (e.g., high school athletic associations, State Departments of Education) are adopting policies that mandate training to equip coaches to support student-athlete mental health (1–3). These policy mandates are especially timely, as high school student-athletes in the U.S. report difficulties balancing academic and athletic demands (4). At the same time, coaches report low confidence in identifying and responding to mental health issues among student-athletes, but they express a strong desire for additional training (5). Hence, targeted, accessible, and scalable trainings are needed to equip coaches with the knowledge, skills, and confidence to recognize mental health challenges and respond appropriately.

In 2021, a coalition of sport leaders launched a community-based participatory research (CBPR) initiative to prepare coaches to “go beyond the X's and O's” and promote positive youth development through sport. This effort coincided with the rising mental health crisis among youth (e.g., student-athletes), statewide advocacy, and policy changes that mandated mental health training for school-based coaches in Ohio. The coalition collaborated to design, pilot, and evaluate an in-person mental health training that met this state policy requirement (6). Preliminary evaluation results from the in-person training were promising; however, additional delivery modalities were needed to implement the policy and reach more coaches statewide. In response, coalition members developed an online mental health training, offering an opportunity to compare the effectiveness of in-person and online modalities in enhancing coaches' confidence and perceived efficacy in supporting student-athlete mental health.

To date, few studies have examined how variations in training delivery methods or pedagogical design shape coach learning, particularly in the mental health domain, creating a gap in understanding how best to build coaches' confidence and competencies (7–9). Existing programs, such as Australia's *Ahead of the Game*, provide in-person, online, and hybrid resources to help parents, coaches, players, umpires, and volunteers recognize and respond to mental health concerns in sport settings (10). In parallel, scholars in Singapore, Portugal, and Canada have investigated the use of online trainings and digital tools to promote positive youth development, especially the teaching of values and life skills through sport (11–14). Across this body of work, evidence suggests that online modalities can enhance coaches' knowledge, reinforce key PYD principles, and serve as an accessible pathway for training delivery. Despite these advances, little is known about how online delivery compares with in-person approaches for developing coaches' confidence and competencies in mental health, or which pedagogical features are most effective in this domain.

Moreover, to our knowledge, no studies have examined the effectiveness of online mental health training in the U.S. context following the enactment of recent state-level policy mandates, nor have they offered guidance on training design, delivery modalities, or content formats to inform future legislation. As such, the purpose of this study was to compare the efficacy of coaches completing online and in-person mental health trainings, and to identify how, if at all, the use of technology, such as specific design features and instructional strategies, via the online delivery influenced coaches' perceived learning.

### Policy landscape: coach mental health training

Around the world, sport systems are increasingly recognizing the critical role coaches can play in supporting youth mental health and well-being. National mental health guidelines in community sport in Australia (15), the United Kingdom's *Mental Health Charter for Sport and Recreation* (16), and the International Olympic Committee's consensus statement on mental health in sport (17) highlight opportunities to equip coaches to recognize distress and intervene when concerns arise. Reflecting similar priorities, recent shifts in U.S. policy highlight how coaches can serve as frontline responders and support youth well-being through sport. Atkinson and colleagues (1–3) documented a rise in mental health training requirements within coaching licensure, credentialing, or permitting processes. As of 2025, nine states passed laws requiring mental health or suicide prevention training for coaches, and several others were considering comparable measures in related oversight bodies (3).

These policy shifts align with and reinforce the evolving priorities of school-based sport leaders and coaches in the U.S. School-based sport leaders recently identified athlete health and safety, mental wellness, and coach professional development as their most pressing concerns (2). Coaches themselves further echoed these priorities, with 67% of respondents in the U.S. National Coach Survey expressing interest in additional mental health training (18). Together, these global trends, policy shifts, and data underscore the growing recognition of and opportunities to better support coaches in identifying and responding to mental health concerns.

### Training modality, content, and instructional design

Correspondingly, an emerging body of research is beginning to shed light on how coaches can be effectively trained in mental health. Several studies indicate that mental health training programs can improve coaches' knowledge, confidence, and ability to refer athletes to appropriate resources (6, 10, 19). Programs such as *Ahead of the Game*, *Coach Beyond*, and *SupportForSport.org*, along with several e-learning mental health modules (6, 10, 20, 21), have demonstrated measurable improvements in mental health literacy or knowledge gains among coach participants. However, despite this progress, important gaps remain in understanding to what extent online delivery can influence coach preparation and whether gains in literacy translate into changes in coaching practice (21).

In the U.S., national trends suggest that training requirements for school-based sport coaches are primarily delivered through online platforms. As of 2025, the majority of state-approved trainings were delivered entirely online (67%), with fewer states offering in-person (6%) or hybrid options [27%; (3)]. State-approved online training requirements often include courses focused on pedagogy [e.g., *Fundamentals of Coaching*; (22)] and other health and safety topics [e.g., concussion, sudden cardiac arrest; (3)]. The COVID-19 pandemic likely accelerated a shift toward online delivery, reflecting a growing recognition of its scalability, especially in reaching U.S. coaches, many of whom do not work in the schools where they coach (1, 23, 24). While online platforms offer convenience, they require intentional design features tailored to adult learners and context-specific examples to promote meaningful skill development.

Several key online features have been shown to enhance coach learning via online learning modules. These include, but are not limited to, reflective journaling (12), establishing relevance, facilitating bridging, designing for personalization, and building community (25). However, Cushion and Townsend (9) argue that the literature on technology-enhanced learning (TEL) should more directly examine changes in coaches' knowledge and focus on how pedagogical design factors influence learning. Notably, Cushion and Townshend's (9) review of TEL did not identify any analyses of online mental health trainings. Building on this gap, Matthews et al. (21) point toward opportunities to examine instructional and pedagogical design elements that can enhance coach competencies beyond mental health literacy alone. Skills such as active listening, empathetic communication, ensuring safety, and making appropriate referrals likely require modeling as well as opportunities for practice, feedback, and reflection. Understanding how to increase coach efficacy in these skills warrants further investigation, especially if more states require coach mental health training.

### Coach education and self-efficacy

Self-efficacy theory (26) is a valuable framework for understanding how coaches can develop confidence and implement new skills. According to Bandura (26), individuals with greater self-efficacy are more motivated to pursue challenging tasks. When considering evolving mental health challenges in sport, strengthening coach self-efficacy may be a critical pathway to improving coach preparation and readiness to identify and respond to mental health concerns. Self-efficacy develops through four pathways: performance accomplishments, vicarious experience, social persuasion, and physiological and emotional feedback (26). Accordingly, effective training programs often include elements such as seeing new skills demonstrated by others (i.e., vicarious experience), practicing skills (i.e., performance accomplishments), receiving feedback (i.e., verbal persuasion), and reflecting upon one's learning [i.e., emotional feedback; (26, 27)]. Because self-efficacy is a key driver of behavior change, training programs that intentionally embed these components are more likely to help coaches adopt and apply new skills.

Feltz and colleagues (28) extended Bandura's theory into sport by outlining key dimensions of coaching efficacy. Building on this foundation, a growing body of research has demonstrated how coach education can predict coaching efficacy (29–31). For instance, Myers (30) found that coach education significantly predicted dimensions of coaching efficacy among a sample of high school coaches. Sullivan et al. (31) similarly identified coaching education as a predictor of efficacy, which in turn predicted leadership behaviors among community and competitive coaches. In a synthesis of the literature, Boardley (32) then noted this established connection but called for additional research examining whether increases in coaching efficacy translate into meaningful behavioral change. Notably, very little research has examined how mental health coach training influences coaching efficacy, and almost no literature has explored to what extent training modality (e.g., in-person vs. online) differentially contributes to efficacy gains. As a result, it remains unclear whether online and in-person formats produce similar perceptions or whether one modality is better suited for building mental health-related skills.

Furthermore, little is known about which specific instructional design features within each modality most effectively support the development of coaching efficacy. These gaps underscore the need to better understand both the impact of modality and the training elements that promote self-efficacy in coaching education, but specifically mental health preparation.

### Current study

Our study aimed to investigate the impact of training modalities on coach self-efficacy in supporting student-athlete mental health. The following research questions guided our study: 1) How do coaches rate their confidence (e.g., efficacy) in supporting student-athlete mental health after completing online vs. in-person training? 2) What key themes emerge from coaches' reported knowledge gains, skill development, and feedback following completion of an online mental health training? Through these questions, we sought to deepen our understanding of how technology can be leveraged to enhance coaching efficacy and improve the quality and design of mental health training for school-based sport coaches.

## Methods

### Context

In 2021, a community-based participatory research (CBPR) initiative in Ohio, co-led by LiFE*sports* at The Ohio State University, the Ohio High School Athletic Association (OHSAA), and sports leaders from 10 school districts, developed *Coach Beyond* (coachbeyond.osu.edu). *Coach Beyond* has established itself as a national leader in coach and student-athlete research, education, training, and support that goes “beyond the X's and O's” (4, 6, 33). One of its key early outputs was a 1-hour training program for coaches focused on supporting student-athlete mental health. This training was based on practical, evidence-informed strategies and has since been implemented in-person for nearly 2,500 coaches in Ohio. Early evaluation results showed significant improvements in coaches' awareness, knowledge, and confidence in helping youth with mental health concerns [see more in (6)].

In July 2023, Ohio House Bill 33 mandated mental health training for all school-based coaches. In response, the Coach Beyond team adapted the in-person training into an online, asynchronous format. At that time, the state agency overseeing policy implementation issued a call for online mental health training programs to meet the new legislative requirements. The *Coach Beyond*: *Supporting Student-Athlete Mental Health* training received approval for the state mandate in 2023 and has since been completed by over 4,500 coaches. It is currently available online nationwide to coaches for $10 through The Ohio State University and for free through the Athletics Healthspace app (34). For this study, secondary de-identified evaluation data from both the online and in-person trainings were analyzed, and all study procedures were approved by The Ohio State University Institutional Review Board (Protocol #2021B0351).

### Online training design

The state-approved 1-hour online mental health training *Coach Beyond: Supporting Student-Athlete Mental Health*, developed by The Ohio State University, was designed in Articulate 360. Developers collaborated with the university's education technology team to design an interactive, accessible training grounded in evidence-based strategies for adult learning. Building upon pilot data and evaluation feedback from in-person mental health trainings, the online training aimed to deepen content, incorporate interactive learning, and use sport-themed gamification to reinforce key concepts and learning. The key objectives guiding the training included: 1) identify the underlying causes of mental health concerns and effects of stress and trauma; 2) recognize the signs and symptoms of mental health concerns; 3) examine techniques to support student-athletes with mental health concerns; 4) identify linkage and referral opportunities to mental health clinicians and services. An overview of the training content is outlined in Table 1 [see (6), for a detailed outline of the in-person training].

**Table 1:** Online training content areas, descriptions, and designs.

Content area	Strategy to build efficacy	Description and design
Definitions and Statistics	Psychoeducation	Key mental health definitions were introduced early to create a shared understanding of terms. National and youth-specific prevalence statistics are included to underscore the urgency and scope of youth mental health issues.
Neurosequential Model of Therapeutics Framing	Psychoeducation	A section on the brain and behavior introduced the “Regulate, Relate, Reason” (36) framework to help coaches apply neuroscience in practical ways and reflect on their behaviors to support youth in engaging in emotional regulation, a key skill underlying mental health risks.
"4 Bs” Examples	Psychoeducation	The original in-person “4 B’s” discussion card deck (Biology, Biography, Behaviors, Backstory) was adapted into clickable examples that illustrate how mental health presents in youth within and beyond the sport context using the “4 B’s.”
Wellness Check-Ins & Brief Mental Performance Strategies	Vicarious experience	Daily wellness check-ins coaches could use were described similarly to the in-person training, and several brief mental performance strategies, such as body scans, quick mindfulness exercises, and deep breathing techniques, were taught using images and voiceovers.
Knowledge Checks	Performance accomplishments	Periodic quizzes or knowledge checks, framed as sport activities, were used throughout the training to reinforce learning and maintain high engagement. An example is a statistic was shown, and the coach had to put the correct soccer ball in the net (e.g., depression, anxiety, or suicidal ideation) to match the statistic.
Coach Reflections	Emotional feedback	A real coach, “Coach Mike,” added a voiceover after every section of the training, demonstrating how he would integrate the content he learned into his practices.
Q.P.P.R. Video Scenarios [adapted from QPR Institute, 2025 (35)]	Vicarious experience, performance accomplishments, and verbal persuasion	The suicide prevention content was expanded with coach-athlete demonstration videos. Coaches were asked to watch 12 videos of a simulated coach and athlete (3 for each element of Question, Pause, Persuade, and Refer; Q.P.P.R) and identify which was a: Strikeout—Coach misses cues or responds poorly (poor) Double—Moderate, supportive response (good) Home Run—Ideal, empathetic, and referral-based response (exemplary)
Practical Tools & Resources	Emotional feedback	The following practical tools were also embedded for downloading during the training: Common signs and symptoms of mental health handout Wellness check-in ideas handout Digital “Make the Call” cards, inclusive of the National Suicide Helpline

Efficacy strategies (26, 27) were defined as: see new skills demonstrated by others (i.e., vicarious experience), practice skills (i.e., performance accomplishments), receive feedback (i.e., verbal persuasion), and reflect upon their experience engaging in the new skill (e.g., emotional feedback). Psychoeducation involved providing coaches with information from credible sources or using examples to detail specific concepts and increase awareness.

### Sample

Since 2023, coaches participating in the mental health online and in-person trainings have had the option to complete an evaluation measure. In total, 1,690 coaches have completed the evaluation for either training: 978 for the online training and 712 for the in-person training. The demographics of the two groups are comparable, with a majority being male, identifying as White, and coaching primarily in school settings. Demographic characteristics of those completing the training evaluations are reported in Table 2.

**Table 2:** Coach demographics (*N* = 1,690).

Demographic Characteristic	Online (*n* = 978)		In-person (*n* = 712)	
	*n*	%	*n*	%
Gender				
Male	588	60.1%	379	53.3%
Female	379	38.8%	327	45.9%
Transgender male	1	.1%	–	–
Non-binary	2	.2%	1	.1%
Self-describe	–	–	2	.3%
Prefer not to answer	8	.8%	3	.4%
Race				
Black/African American	78	8.0%	50	7.0%
Hispanic/Latino	10	1.0%	8	1.1%
Asian/Pacific Islander	5	.5%	5	.7%
White	836	85.5%	616	86.6%
Native American or American Indian	6	.6%	1	.1%
Multiple Races	15	1.5%	14	2.0%
Other/Prefer not to answer	28	2.9%	18	2.5%
Teacher or Staff Member at a School				
Yes	469	48%	469	65.9%
No	500	51.1%	235	33.0%
Did not answer	9	.9%	8	1.1%
Coach Setting				
School	777	79.5%	563	79.1%
Developmental (8 years or younger)	8	.8%	3	.4%
Recreational	21	2.1%	28	3.9%
Competitive	130	13.3%	86	12.1%
Adapted setting	2	.2%	–	–
Other setting	40	4.1%	32	4.5%
Top 6 Sports Coached^a^				
Football	141	14.4%	118	16.6%
Basketball—Girls	121	12.3%	65	9.1%
Basketball—Boys	111	11.3%	51	7.2%
Track & Field—Girls	101	10.3%	55	7.7%
Track & Field—Boys	92	9.4%	46	6.5%
Volleyball—Girls	87	8.9%	49	6.9%

a Represents the top six sports; therefore, percentages and respondent counts do not total 100%.

### Evaluation measures

#### Learning objectives

A brief retrospective pre-post-test evaluation survey was designed [see (6)] to assess the learning objectives of the in-person and online mental health trainings. Questions were presented in the evaluation survey with different stems (i.e., “Before/After attending this training, how confident did/do you feel in your ability to”) to capture changes in efficacy over time. All items were measured on a five-point Likert-style scale (1 = *Not at all confident*; 5 = *Extremely confident*). Both the online and in-person modalities evaluated coaches' perceptions of their ability to (1) “support student-athletes with mental health concerns” and (2) “link student-athletes to mental health supports in their school or community.”

#### General evaluation of training

Coaches also rated their agreement with several general evaluation statements on a five-point Likert scale (1 = *Strongly disagree*; 5 = *Strongly agree*). Such statements included: (1) *I see value in paying attention to student-athletes' mental health*; (2) *I am more prepared to support student-athletes with mental health concerns*; (3) *I would recommend this session to a friend or colleague*; (4) *I thought the instructor(s) were knowledgeable about this topic*; and (5) *I am interested in learning more about this topic*.

#### Open-ended questions

Additionally, the evaluation survey for the online training included two open-ended responses: “*What is one thing you learned from today's session that you will use in the future?*” and “*Please provide any additional feedback on today's session here*.”

### Data analysis

The inclusion criteria for data analysis included having complete demographic information and (1) answering at least one set of matching Likert-style evaluation questions (e.g., before and after) or (2) providing at least one open-ended response in the evaluation. Based on these criteria, fewer than 20% of cases were removed using listwise deletion, and sample sizes for each question are reported to detail the number of respondents per item. All analyses were conducted using IBM SPSS Statistics (Version 29). Descriptive statistics (e.g., means and frequencies) were calculated to summarize confidence levels across time points (e.g., before and after) and by delivery modality (e.g., online and in-person).

To examine changes in coach confidence over time by modality, a series of repeated measures ANOVAs was conducted on two items gauging coaches' perceptions of their ability to: (1) support student-athletes with mental health concerns, and (2) link student-athletes to mental health supports in their school or community. Each model included time (pre-training and post-training perceptions) as a within-subjects factor, and training modality (online vs. in-person) as a between-subjects factor. Effect sizes were reported using partial eta squared (*η*^2^), with values of .01, .06, and .14 interpreted as small, medium, and large effects, respectively (37). An alpha level of *p* < .05 was used to determine statistical significance.

In addition, researchers analyzed open-ended questions using conventional content analysis (38). Two research team members independently read the data repeatedly to establish familiarity. Then, each researcher manually developed initial codes for each question. The most frequent responses to each question were included to highlight the top descriptions of what coaches learned and their overall feedback on the online training design.

## Results

### Quantitative results

#### Learning objectives

Descriptive statistics revealed that both groups experienced gains in self-reported confidence over time (see Table 3). Among online participants, the percentage who reported being moderately or extremely confident in supporting student-athletes with mental health concerns increased from 44% before training to 94% after training (Mean Δ =  + 1.13). Similarly, confidence in linking student-athletes to mental health supports increased from 42% to 89% (Mean Δ =  + 1.20). Among in-person participants, the percentage who reported high levels of confidence in supporting student-athletes with mental health concerns increased from 39% before training to 88% after training (Mean Δ =  + 0.95). Similarly, confidence increased from 39% before training to 89% after training among in-person participants when assessing their confidence in linking student-athletes to mental health supports (Mean Δ =  + 1.06).

**Table 3:** Perceptions of coach efficacy before and after mental health training by modality.

Training modality	Evaluation Item	Before…		After…	
		Moderately or extremely	Mean (SD)	Moderately or extremely	Mean (SD)
Online (*n* = 935)	Support student-athletes with mental health concerns.	44%	3.34 (1.01)	94%	4.47 (.63)
	Link student-athletes to mental health supports in your school or community.	42%	3.21 (1.01)	89%	4.41 (.72)
In-person (*n* = 679)	Support student-athletes with mental health concerns	39%	3.26 (.94)	88%	4.21 (.67)
	Link student-athletes to mental health supports in your school or community.	39%	3.25 (1.02)	89%	4.31 (.69)

### Time and delivery modality

A repeated measures ANOVA revealed a significant main effect of time (perceived efficacy before and after training), *F*(1, 1637) = 2344.67, *p* < .001, *η*^2^ = .589, indicating that coaches significantly increased their general efficacy in supporting student-athletes after completing the training, regardless of delivery modality. There was also a small but significant time × modality interaction, *F*(1, 1637) = 17.38, *p* < .001, *η*^2^ = .011. While both groups experienced statistically significant increases in efficacy, the online group showed a slightly greater improvement (Mean Δ =  + 1.13) than the in-person group (Mean Δ =  + 0.95), although the effect size was small.

A second repeated-measures ANOVA examined changes in coaches' confidence in linking student-athletes to mental health support. A significant main effect of time was observed, *F*(1, 1653) = 2243.93, *p* < .001, *η*^2^ = .576, again reflecting an increase in efficacy from pre- to post-training. A time × modality interaction was also significant, *F*(1, 1653) = 8.96, *p* = .003, *η*^2^ = .005, indicating that the online group experienced a slightly greater increase than the in-person group. Though statistically significant, the effect sizes were small. For both analyses, the between-subjects comparison revealed no significant differences between modalities.

### General evaluation of training

In addition to gains in confidence, participants in both training modalities reported positive perceptions of the training experience (see Table 4). Nearly all participants agreed or strongly agreed that the training helped them see value in paying attention to student-athletes' mental health (96%–97%), feel more prepared to support student-athletes with mental health concerns (96%–97%), and that they would recommend the training to others (94%–95%). Instructor knowledge was rated very highly across both modalities (96%–97%), and a large majority of participants (85%–86%) indicated interest in learning more about the topic.

**Table 4:** Perceptions of training effectiveness by modality.

Evaluation Question	Online			In-Person		
As a result of attending this training…	*n*	Somewhat or strongly agree	Mean (SD)	n	Somewhat or strongly agree	Mean (SD)
I see value in paying attention to student-athletes’ mental health.	931	97%	4.86 (.54)	677	96%	4.82 (.54)
I am more prepared to support student-athletes with mental health concerns.	932	97%	4.70 (.62)	678	96%	4.68 (.59)
I would recommend this session to a friend or colleague.	932	94%	4.67 (.67)	678	95%	4.73 (.62)
I thought the instructor(s) were knowledgeable about this topic.	932	96%	4.79 (.56)	676	97%	4.89 (.44)
I am interested in learning more about this topic.	932	85%	4.41 (.82)	678	86%	4.42 (.78)

### Qualitative results

#### Skills and future application

Approximately 856 comments were analyzed and clustered into themes to capture participants' reflections on what they learned from the training and how they will use it in the future. Many coaches (*n* = 200) reflected on learning how to approach struggling athletes, highlighting new skills in de-escalation and emotional regulation that they had acquired from the modeled difficult conversations in the videos. One participant specifically noted, “One of the biggest take away from me is learning how to frame these difficult conversations with student-athletes from watching [coach's] videos.” Other participants shared that, “The modeled conversations with athletes and phone calls to parents/counselors were really helpful. I will apply some of the sentence starters to my conversations with my athletes.” Additionally, participants reported learning strategies to check in with student-athletes, including daily relational strategies to monitor their well-being (*n* = 148). Some coaches highlighted specific check-in strategies, such as “Check-in tools like the bus or the sport-specific analogies that get folks talking in less threatening ways.”

Third, participants (*n* = 140) learned about Q.P.P.R. (Question, Pause/Persuade, Refer), which enhanced coaches' recognition of warning signs and increased their confidence to engage in crisis conversations. In the words of one coach, “I will use that QPR techniques with my student-athletes to help them navigate mental and physical demands of life and sport.” Within this domain, participants reported learning how to create dialogue with open-ended questions (*n* = 135). One coach revealed, “I learned how to phrase questions to elicit better and more in-depth responses,” while another reflected that, “Using open-ended questions will help students answer more truthfully or thoughtfully.” Finally, coaches reported learning the importance of referring student-athletes to resources (*n* = 89), underscoring the need to know referral pathways for athletes. One coach shared, “I'm going to keep a list of the school resources and city resources in my coaches bag!,” demonstrating preparation to utilize referral resources when needed.

### Feedback on training

In total, approximately 464 comments were analyzed and clustered into several themes to reflect participants' feedback about the online training. Overall, the feedback was positive (*n* = 159), with participants frequently using descriptors such as “great,” “fantastic,” “well done,” and “helpful.” Many emphasized that the session was enjoyable, worthwhile, and insightful. Participants consistently described gaining new knowledge or reinforcing prior understanding of mental health topics. Several even shared personal stories of struggling with or supporting others with mental health concerns. Second, participants commented that roleplays and scenario-based learning were the most valued elements of the training (*n* = 43), specifically highlighting the utility of online activities, case studies, and example videos. These scenarios were described as practical, realistic, and effective in modeling best practices. Several noted that the contrast between poor, good, and exemplary approaches was beneficial. Third, coaches commented on the training's applicability (*n* = 53) by describing how they planned to implement check-ins, use communication strategies with athletes and parents, and integrate mental wellness activities into team routines.

A smaller number of coaches described issues with the training, such as the cost of access (*n* = 4), advocating that the training should be free for volunteer coaches, incorporating it into mandatory certification requirements, or requiring it for all coaching staff. Another subset of coaches (*n* = 22) expressed a desire for additional materials, follow-up courses, or in-depth exploration of specific topics. Finally, coaches described ways the training could be refined, with several noting technical problems (*n* = 22; e.g., multiple play buttons, platform glitches, slow pace). Others noted a desire for more straightforward navigation, such as the ability to rewind videos more fluidly. Others critiqued specific activities or examples (*n* = 12). For instance, some felt exercises lacked realism, or that specific simulations did not reflect everyday coaching situations. A few expressed dislikes for the added “coach commentary” segments.

## Discussion

The current study sought to evaluate how coaches rated their efficacy in supporting student-athlete mental health after completing online vs. in-person training, and to examine the specific themes that emerge in coach feedback on the knowledge and skills gained from online mental health training. Our findings indicated that both training formats were effective in increasing coaches' efficacy in addressing mental health concerns and linking student-athletes to mental health resources. The effectiveness of this training may be supported by the CBPR nature of the project [see (6)], as a group of coaches and athletic directors informed the training curricula and advised developers about the intentional need to utilize interactive, sport-specific, and applied learning activities. Embedded in both modalities, the content was designed to align with adult learning strategies (e.g., knowledge checks) and those shown to support the development of coach efficacy (e.g., modeling, opportunities for reflection). Additionally, both modalities were tailored to a coaching audience. For example, in the in-person training, the “4 B's” discussion deck uses sport-specific examples, making each card an awareness-raising activity coupled with psychoeducation (6). Whereas in the online training, developers used voiceovers and videos of coaches to model reflections and sport examples, scenarios, and activities that model interpersonal skills necessary for engaging in behaviors that support identifying and responding to mental health concerns.

Regarding coach efficacy, our results indicate that online participants demonstrated marginally greater gains across both efficacy domains. These differences, while statistically significant, were small in effect, suggesting that online delivery may offer a slight added benefit in enhancing coaches' efficacy. One reason coaches may have perceived greater gains in the online format is denoted in our qualitative findings. Qualitative results reveal the interpersonal skills and strategies that coaches acquired from the training, such as conducting wellness check-ins, using Q.P.P.R., and approaching struggling athletes. These reflections suggest that coaches valued learning actionable, relationship-centered strategies that they could immediately integrate into practice. This aligns with, but builds upon, the qualitative findings from prior studies examining pilot sessions of the in-person mental health training, which emphasized asking open-ended questions, along with general comments about increased mental health knowledge and awareness (6). Furthermore, themes of skills learned go beyond literacy and showcase the behaviors coaches can engage in to take action to support a student-athlete who is struggling (21). In prior reviews, the connection between online learning activities and knowledge or skill gains in online coaching education platforms was lacking (9, 32). Our results suggest the videos modeling and requiring coaches to identify poor, good, and exemplary interpersonal skills (e.g., vicarious experience, practice skills, and emotional feedback) with an athlete may have been the most crucial to supporting gains in efficacy. One could argue that perhaps the psychological safety of reviewing and reflecting online may help coaches feel more prepared to apply skills in practice.

Qualitative feedback on the training design highlights several key considerations for the ongoing development of online coach training. First, the positive responses to the design suggest that coaches find online formats acceptable and impactful. The strong emphasis on roleplays, case studies, and scenario-based learning highlights the importance of applied components that simulate realistic interactions and model best practices. At the same time, feedback on cost, accessibility, and integration into certification requirements highlights the barriers that affect coaches' access to continued professional development. As countries and states consider passing policies to support mental health training for coaches, they must also consider the unintended consequences (e.g., increased time and costs, and potential retention issues) and work to address barriers to accessibility. Finally, coach feedback on topics such as fixing glitches and making scenarios more realistic showcases opportunities for continuous improvement in ensuring that online training remains relevant, user-friendly, and responsive to coaches' needs.

### Implications & future research

This study had several meaningful implications for the use of technology in coach education and for informing practice for sport leaders, researchers, policymakers, and coach developers. Our findings support the adoption of high-quality, interactive online mental health training as a scalable approach to engage coaches, particularly in rural, time-constrained, or resource-limited contexts where in-person options may be less feasible. As policymakers internationally and across the U.S. consider implementing mental health training within coaching licensure and permit requirements, online delivery may be a suitable option for scaling education and providing low-cost policy implementation. Based on our findings, we argue that online mental health trainings should include realistic, translational activities and content that support learning and improved efficacy. One caveat is that opportunities for coaches to ask context-specific questions or reflect on their own processes are lost when the training occurs online. For example, the in-person training modality allows a school or community mental health specialist to attend, introduce themselves to coaches, and share referral processes. This is a notable limitation of the online format, as context-based discussions and connections may strengthen the pathways for coaches to link athletes to trained professionals.

Furthermore, technology may help reach more community-based coaches (e.g., those who do not work as teachers or school staff). In our study, 51% of online participants were community-based coaches (e.g., not teacher educators) compared to 33% of in-person participants. Community-based coaches likely have other time demands (e.g., jobs, families, other obligations) or work in contexts where resources to provide professional development are more limited. Prior research indicates that community-based coaches report feeling less efficacious in areas such as mental health and other positive youth development topics than coach-educators [e.g., those working in schools who also coach; (33)]. By removing logistical barriers and allowing for flexible participation, online training can expand the reach of mental health education to coaches who might otherwise be difficult to engage. Leveraging technology to deliver high-quality continuing education for coaches, particularly on skills that extend beyond the “X's and O's,” may play a pivotal role in advancing policy implementation, raising awareness, reducing stigma around mental health in sport, and shaping coach expectations, knowledge, and ultimately behaviors that support student-athlete well-being.

### Limitations

While this study benefited from large sample sizes that strengthened our analyses, our results also warrant caution in interpretation. Large samples can increase statistical power, making even a minimal difference statistically significant, which may not always reflect real-world impact. Additionally, the delivery format introduces potential limitations: in-person sessions were led by different trainers to reach a larger number of coaches, introducing variability in fidelity. Although facilitators completed a standardized train-the-trainer process (e.g., training on content, observation, feedback, and ongoing consultation), the in-person format still allows for some variation in delivery, particularly when responding to context-specific examples or participant questions. In comparison, the online training was standardized in its delivery every time. To strengthen future research, standardization strategies such as fidelity-monitoring checklists and periodic observation with inter-rater agreement could help reduce this variability and allow clearer attribution of effects to in-person training.

It is also possible that participants who chose to complete the evaluations were more favorable toward the training, potentially skewing results in a positive direction. However, because the training was state-mandated, participation in the evaluation may be higher and more representative than in a voluntary training setting. Future research should move beyond self-reported post-training perceptions of efficacy to examine whether gains translate into observable and sustained changes in coaching practice (32). While our findings indicate that both formats are practical and that online delivery offers a scalable option for reaching more coaches, it remains unclear whether these differences persist over time or have a meaningful impact on team environments and student-athletes' help-seeking behaviors. Future studies should also investigate whether training formats influence coaches' behavior and whether these behaviors benefit student-athletes. Randomized, longitudinal, and mixed-method approaches can also provide deeper insight into the extent to which format-specific outcomes are sustained and lead to measurable improvements in student-athlete well-being and access to mental health supports.

## Conclusion

Legislative momentum and international sport leaders continue to elevate the role of coaches in supporting student-athlete mental health. As the U.S. continues to see an uptick in state-level mandates moving beyond policy into implementation, questions around scalability and instructional design for mental health trainings will become increasingly important. This study contributes to that conversation by demonstrating that an interactive, intentionally designed online mental health training program can foster gains in coach efficacy comparable to those achieved through in-person training. Notably, this training was unique in that it was co-developed with a coalition of sport leaders and utilized pedagogical strategies they advocated would enhance learning, particularly in relation to the complex interpersonal skills required to respond to a student-athlete in crisis. Features such as modeling through video demonstrations helped coaches conceptualize and reflect on skill application. While further inquiry is needed to assess whether gains in efficacy lead to long-term behavioral changes, these findings offer a promising direction for leveraging technology to prepare coaches to support student-athlete mental health.

## Data Availability

The raw data supporting the conclusions of this article can be made available upon request from the corresponding author.
